# A Scourge Returns: Black Lung in Appalachia

**DOI:** 10.1289/ehp.124-A13

**Published:** 2016-01-01

**Authors:** Carrie Arnold

**Affiliations:** Carrie Arnold is a freelance science writer living in Virginia. Her work has appeared in *Scientific American*, *Discover*, *New Scientist*, *Smithsonian*, and more.

Once a month, a group of men in t-shirts, jeans, and baseball caps gather around a long table at the New River Health Clinic. The clinic, a small, one-story yellow clapboard building, is located in the tiny town of Scarbro, nestled in the bituminous hills of southern West Virginia. The members of the Fayette County Black Lung Association greet each other by name while they pour bitter black coffee into small Styrofoam cups.

**Figure:**
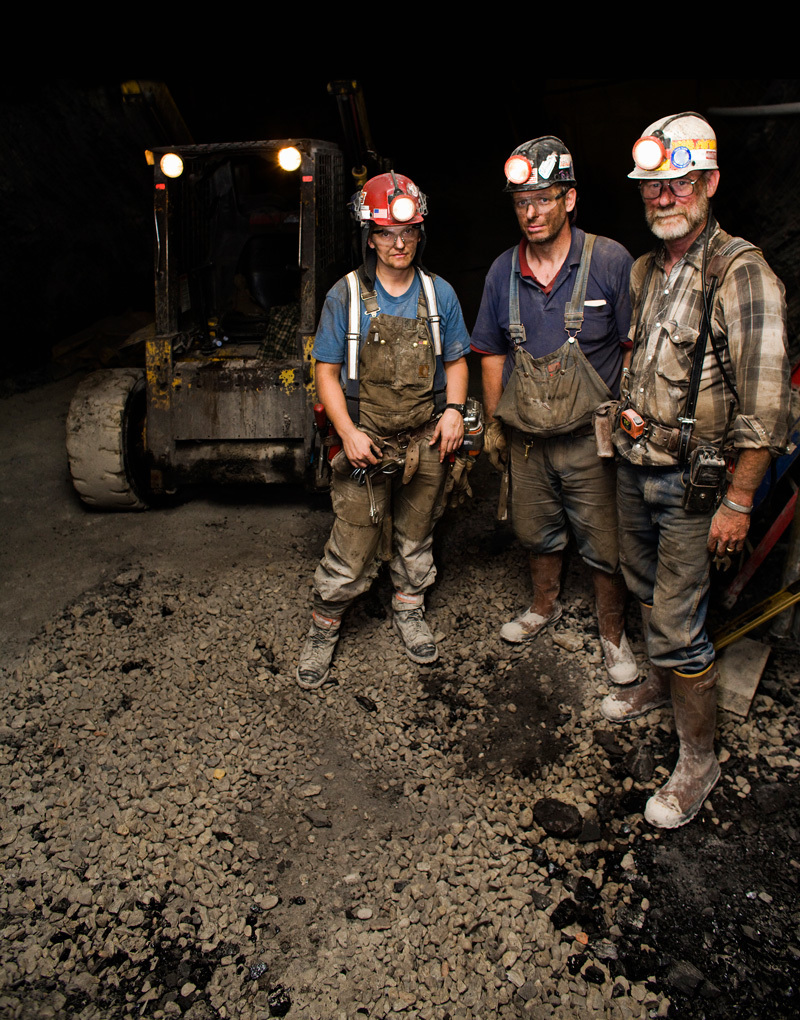
In the early 1970s, coal workers’ pneumoconiosis, or black lung, affected around one-third of long-term underground miners. After new dust regulations took effect, rates of black lung plunged. Today, however, they are once again rising dramatically, and the new generation of black lung patients have disease that progresses far more rapidly than in the past. © Tyler Stableford/Getty

Amidst the chatter and the coffee are the coughs. Some of the men hack loudly, others more quietly. All of them have advanced black lung, a disease they acquired working in the local mines. Although roughly 22% of underground miners smoke,^1^ compared with about 18% of U.S. adults in general,^2^ none of these men do. They gather not just as a support group but also to help one another complete the stacks of paperwork necessary to apply for government-mandated benefits for black lung and navigate the tortuous appeals process.

Aside from the group’s leader, a bespectacled septuagenarian named Joe Massie, all the other members are in their 50s or early 60s. That’s relatively young for someone with advanced black lung, and other workers are getting sick even earlier. These miners, who have gotten so sick so fast, are on the forefront of a wave of new black lung cases that are sweeping through Appalachia.

Scientists first noticed a troubling trend in 2005, when national surveillance conducted by the National Institute for Occupational Safety and Health (NIOSH) identified regional clusters of rapidly progressing severe black lung cases, especially in Appalachia.^3^ These concerns were confirmed in followup studies using a mobile medical unit providing outreach to coal mining areas,^4^^,^^5^ with later research showing that West Virginia was hit particularly hard.^6^ Between 2000 and 2012, the prevalence of the most severe form of black lung rose to levels not seen since the 1970s,^7^ when modern dust laws were enacted.^8^

Scarier still, the new generation of black lung patients have disease that in many cases progresses far more rapidly than in previous generations. Today, advanced black lung can be acquired within as little as 7.5–10 years of beginning work, says Edward Petsonk, a pulmonologist at West Virginia University. But not all cases progress so quickly; thus, occupational health researchers fear that what they are seeing now is only the tip of the iceberg.

## The History of Black Lung

Black lung is not a new disease. Ever since humans first started mining coal nearly 5,000 years ago in Bronze Age China,^9^ those who worked in the mines breathed in the black dust that, over time, destroyed their lungs.

Writing in 1846, Scottish physician Archibald Makellar sketched out the course of the disease in miners exposed to extremely high levels of dust: “A robust young man, engaged as a miner, after being for a short time so occupied, becomes affected with cough, inky expectoration, rapidly decreasing pulse, and general exhaustion. In the course of a few years, he sinks under the disease; and, on examination of the chest after death, the lungs are found excavated, and several of the cavities filled with a solid or fluid carbonaceous matter.”^10^ Makellar called the disease “black phthisis.” Later physicians gave black lung its official modern name of coal workers’ pneumoconiosis (CWP).

The disease starts with dust—whether swinging picks or using large machines, the process of breaking up coal and extracting it from its prehistoric home creates vast amounts of dust. And unless effective measures are used to control airborne dust in the narrow underground shafts, miners can breathe it into their lungs.

Coal mine dust isn’t uniform; it’s a jumble of substances and particle sizes, which vary in their effects on the lungs.^11^ Larger “thoracic” particles settle in the bronchi, the main air passages to the lungs.^12^ The presence of coal mine dust in the bronchi stimulates the production of mucus, Petsonk explains, so that people can more easily cough up the offending particles. It’s an efficient system, but prolonged inhalation of the dust can lead to chronic bronchitis in miners. “Coal dust particles are very reactive, including the chemical bonds on the surface,” Petsonk explains. “They will interact with anything nearby, including the body’s tissue, which creates an inflammatory response.”

It’s the smaller respirable dust particles, though, that create the damage most associated with CWP. Because of their small size—often 2.5 microns or less in diameter—they can easily travel beyond the bronchi, into the bronchioles and alveoli. Any small particle this deep in the lungs, whether from cigarette smoke, car exhaust, or coal mine dust, can create irritation in the site where it lands.^13^ The body’s immune system attacks the particles, creating inflammation in the surrounding region. Although this inflammation can help kill invading pathogens, it can’t remove components of coal mine dust such as coal and silica, which remain in place and cause lung tissue damage. The body then doubles down on its efforts, which further damages the delicate lung tissue. The result is chronic inflammation that ultimately scars the lungs, creating patches that radiologists can see on X rays and CT scans.^14^

**Figure:**
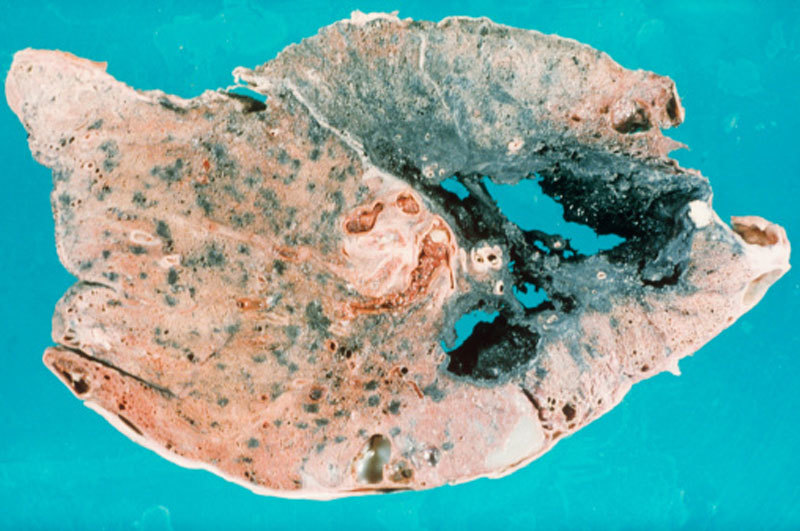
A section of lung shows the ravages of progressive massive fibrosis (PMF). The disease is characterized by large, dense masses of fibrous tissue that often appear in the upper lungs. The lung itself can appear black due to the slow buildup of coal dust particles over the years. © Biophoto Associates/Getty

**Figure:**
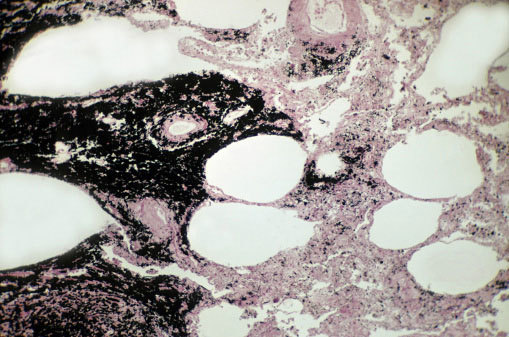
Inset: Highly reactive particles of coal mine dust can infiltrate the deepest reaches of the lung. These inhaled particles of coal dust and/or silica create a chronic inflammatory response that damages the lung. © Ed Reschke/Getty

Smaller patches of damage may have relatively little effect on a miner’s lung function measurements. Over time, however, the damage becomes more widespread, creating the 1- to 2-mm nodules of immune and inflammatory cells, collagen fibers, and black dust indicative of so-called simple CWP.^15^ Symptoms of simple CWP include chronic cough, increased phlegm production, and shortness of breath. CWP sufferers also are at increased risk of emphysema,^16^ which is an important cause of morbidity among miners.^17^

In some patients, the disease progresses to complicated CWP, a condition also known as progressive massive fibrosis (PMF). As its name suggests, PMF is characterized by large, dense masses of fibrous tissue more than 1 cm in diameter, which often appear in the upper lungs.^18^ The lung itself often appears blackened. The presence of fibrosis impairs the ability of the lungs to bring oxygen to the blood, which leaves sufferers chronically short of breath and may result in death.^6^

Initially, coal dust itself was seen as rather harmless, and the true cause of CWP was believed to be silicosis. This disease is caused by inhaling particles of respirable crystalline silica, which also can be found in coal mine dust.^19^ Indeed, the symptoms of CWP overlap with those of silicosis; the two diseases can look similar on X rays, and both fall within the constellation known as coal mine dust lung disease.^18^^,^^19^ However, work begun in the nineteenth century by Makellar^10^ and fellow Scottish physician J.C. Gregory,^20^ which continued into the 1920s and ’30s, began to focus specifically on coal dust as the sole culprit of CWP.^21^^,^^22^ By the 1950s, scientists had shown with near certainty that CWP could be caused exclusively by excessive exposure to coal dust.

This came as no surprise to the tens of thousands of coal miners working throughout Appalachia and across the rest of the country, who for decades had observed and experienced the devastation caused by black lung. By the late 1960s, the crisis had come to a head. In 1968 the members of United Mine Workers of America went on strike to create better working conditions, including protection from coal mine dust, and to set up a fund for miners disabled by black lung.^23^

The strike worked. In 1969 Congress passed the Federal Coal Mine Health and Safety Act, or Coal Act for short, which was signed into law by President Richard Nixon.^24^ The Coal Act created the agency that would become the Mine Safety and Health Administration, and required that every underground coal mine be inspected four times per year and surface mines twice a year. The Act also set limits on the amount of dust that miners could be exposed to and developed procedures for miners disabled by CWP to receive compensation.

In the early 1970s, shortly after the Coal Act went into effect, CWP affected around one-third of miners who had worked underground for more than 25 years.^5^ As the new rules and regulations took effect, rates of CWP began to drop, then plunge. By the 1990s, it seemed CWP was on its way to becoming a thing of the past.^25^

## Fighting the Dust

Another requirement of the Coal Act was the creation of the Coal Workers’ Health Surveillance Program (CWHSP), a voluntary screening program for black lung in which miners receive X rays upon hiring and then can return for followup every five years thereafter. One of the physicians responsible for evaluating those X rays was Petsonk. After the number of miners diagnosed with CWP began to drop in response to the improved dust standards,^25^ Petsonk expected they would keep dropping—except they didn’t. In the early 2000s, Petsonk believed he was seeing an increase in the number of PMF cases, but he needed data to back up his perception.

In 2005 he and other NIOSH investigators published the initial evidence of geographical clusters of rapidly progressing cases of CWP, including in Appalachia.^3^ In 2011 Petsonk and colleagues published a study of 138 West Virginia miners compensated by the state for PMF between 2000 and 2009.^6^ All those miners had spent their careers in the mines long after the Coal Act went into effect. The study thus indicated that either the Coal Act standards were not adequate or the rules were not being followed, or both.

**Figure:**
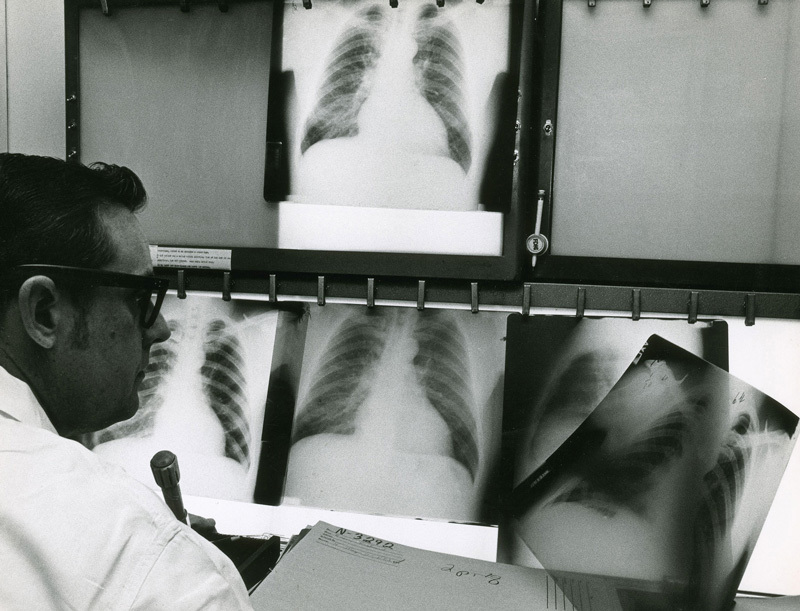
The Coal Workers’ Health Surveillance Program was created in the early 1970s under the Coal Act. Miners who participate in the voluntary program receive an X ray upon being hired, then may return for followup X rays every five years. In the mid-2000s, doctors participating in this program alerted federal authorities to the resurgence of black lung among coal miners in Appalachia. © Michael Sullivan/Science Source

“The only thing that causes this illness is the inhalation of dust during coal mining,” says David Weissman, director of the Respiratory Health Division at NIOSH. “To have people getting sick so young, they must have been way overexposed, which means failures in [regulatory] compliance.”

The black lung data coming in from NIOSH’s screening programs indicated that the rise in CWP was most severe in Kentucky, Virginia, and West Virginia,^26^ and that miners working in small operations (fewer than 155 miners) were more likely to be affected than those from larger outfits.^27^ Compared with miners in other states, these miners were also younger, had worked in underground mines for fewer years, and were more likely to have PMF, the most severe form of black lung.^27^ Another study indicated that abnormal lung function, as measured by spirometry, was three times more prevalent than CWP, suggesting that CWP was not the only disease affecting miners’ lungs.^1^

The screening program is voluntary, and because less than one-third of miners are estimated to participate.^28^ NIOSH researchers conducted further analyses to test the robustness of their initial results. The results of these analyses, reported in 2014, indicated that the original estimates of CWP prevalence among coal miners likely did not overstate and may in fact have understated the true prevalence of black lung.^29^

PMF had become nearly nonexistent in 2000, affecting only 0.08% of CWHSP participants and 0.33% of miners who had worked at least 25 years belowground.^7^ But as investigators from NIOSH and the Centers for Disease Control and Prevention mapped the prevalence of PMF moving forward, they found a steep U-shaped curve. By 2012, they reported, the prevalence had jumped 900% compared with 2000, affecting 3.23% of miners with 25-plus years of work.^7^ “These were levels we hadn’t seen since the early 1970s, shortly after modern dust control measures came into effect,” says coauthor David Blackley, an epidemiologist at NIOSH.

For the NIOSH scientists, perhaps the most frustrating part of seeing these numbers was knowing it didn’t have to be this way. “Coal workers’ pneumoconiosis is an entirely preventable disease. They wouldn’t have gotten sick without inhaling way too much coal dust,” says NIOSH coauthor A. Scott Laney.

To the miners of the Fayette County Black Lung Association, the resurgence was no mystery. According to Terry Lilly, a tall, broad-shouldered miner with a gray mustache, weakening of the coal miners’ union in the 1980s undermined the protections put in place by the Coal Act, leaving workers vulnerable. Lilly, who worked as a foreman, says coal mine officials instructed him to alter measurements of dust levels in the mines.

“We knew when the mine inspectors were coming before they even set foot belowground. They’d call us and let us know we had a visitor, and we’d get to work. We’d place dust monitors below vents, where they’d constantly get fresh air. We’d throw up curtains,” Lilly says. And when the dust got too thick, his choice was to continue working or lose his job. Several decades ago, when the unions were stronger, he’d felt empowered to halt operations in unsafe situations, he says, but those days were long gone.

The mining companies under which the violations occurred have been sold or gone bankrupt, and representatives were unavailable to comment for this story. However, says Luke Popovich, a spokesman for the National Mining Association, “No one in this industry wants a mining accident and consequently do not tell their employees to ignore safety standards for any reason.”

Jason Hayes, associate director for the American Coal Council, adds, “I can’t comment on any anonymous reports. However, I will note that there are very clear federal and state regulations governing respirable dust levels in mines and the safety measures that are required to reduce employee exposure to dust. All American mines are required to follow those regulations.”

## A New Era in Safety?

Many of the largest coal seams were long ago depleted, leaving only smaller, narrower seams for modern-day miners. There is still plenty of coal there, says Lilly, but to make room for the large machinery needed to extract it requires blasting through not just coal but also the surrounding rock. A major component of that rock is silica, the major contributor to silicosis.^30^^,^^31^

**Figure:**
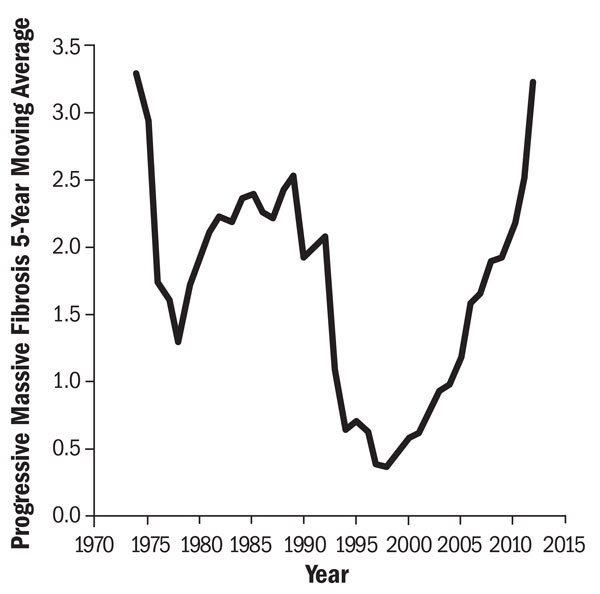
In 1974, shortly after the Coal Act went into effect, PMF affected nearly 3.5% of coal miners with 25 or more years of underground mining tenure. Rates dropped precipitously under the new protective rules but have since rebounded, shooting up 900% over the past 15 years. Source: Blackley et al. (2014)^7^

Weissman and others believe the combination of silica and coal dusts is especially toxic and is helping drive the surge in new CWP cases and causing them to progress so much faster than in previous generations.^31^^,^^32^ “This dust is more toxic, and the miners are inhaling more of it,” Weissman says. Rather than straight CWP, he says, what seems to be developing is a mixed dust disease with the worst aspects of both CWP and silicosis. This idea is supported, he says, by a recent histopathologic analysis of lung samples obtained from coal miners with advanced cases of CWP.^32^

As for why the increase in CWP is most striking in central Appalachia, the content of the coal itself might play a role, says Andrea Harrington, a postdoctoral research scientist at New York University School of Medicine. The coal mined in this region has an unusually high concentration of pyrite, an iron compound commonly known as fool’s gold.^33^ The iron in pyrite is fairly chemically reactive, stripping electrons from water molecules and creating reactive oxygen species in the form of hydrogen peroxide, hydroxyl radicals, superoxide, and/or singlet oxygen.^34^

A 2005 study reported an association between the pyrite content of the coal in various mining regions and the rates of CWP documented there.^35^ Harrington’s work shows that pyrite in coal dust increases inflammation,^36^ which increases lung damage.^37^ “It’s a dose issue,” she says. “Your body can only handle so many particles before it gets overwhelmed.”

The inhalation of any dust is likely to cause issues, Harrington adds. However, adding a reactive metal to inhaled dust only exacerbates the problems.

Decreasing numbers of U.S. coal mines—a result of competition from natural gas and declining profits^38^—means that U.S. coal production also is going down, in 2013 falling below 1 billion short tons for the first time in 20 years.^39^ Pressure to increase productivity with fewer miners means increased mechanization, which results in smaller and thus more harmful dust particles than hand labor can produce. Coal miners have also been working longer hours—this means they not only are exposed for longer periods but also have less time between shifts to clear the dust from their lungs.^29^

The miners believe that, whatever the cause for the increase, stronger labor laws and dust protections will help keep their fellow miners from getting sick. Their desire came to pass in 2014, when the Mine Safety and Health Administration issued updated rules for dust exposure. Among provisions set to go into effect in 2016, the allowable overall dust level was tightened from 2.0 mg/m^3^ to 1.5 mg/m^3^, and mine operators are now required to continuously monitor dust levels and take immediate action if dust levels are high.^40^

**Figure:**
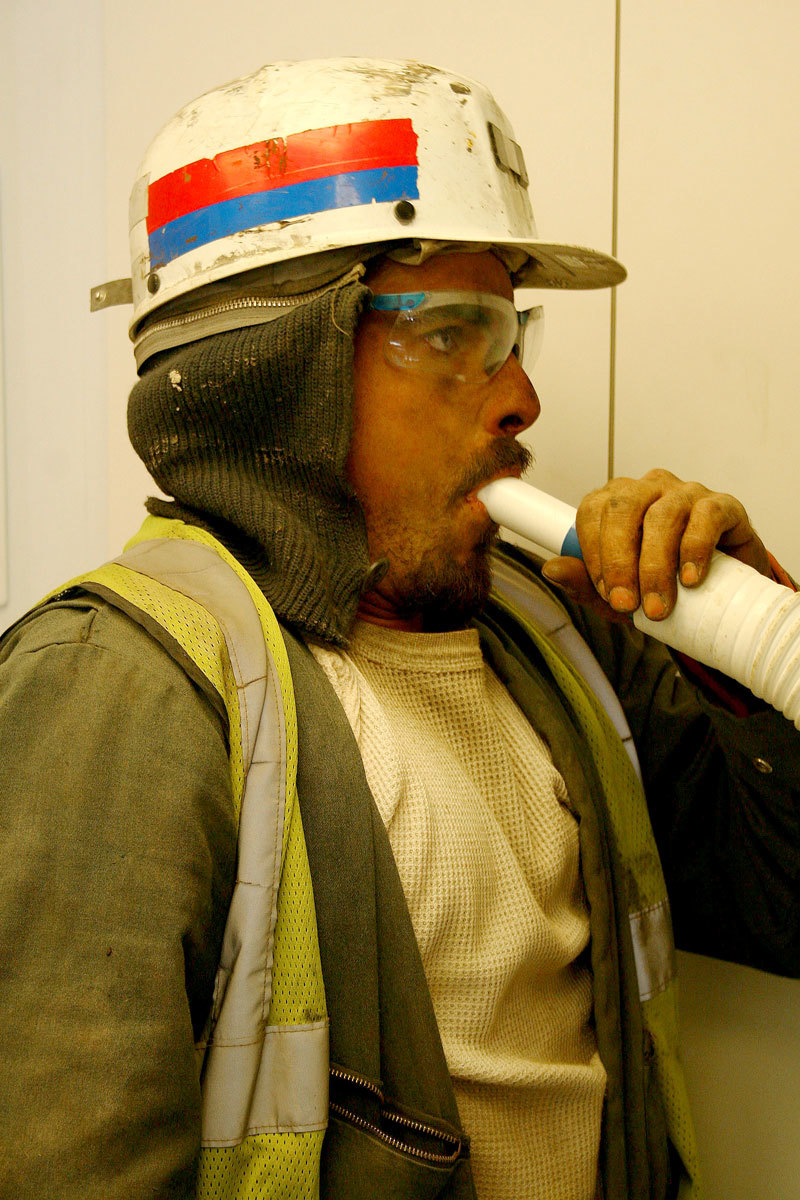
Updated rules for dust exposure call for lung function tests using spirometry, which may help identify other work-related lung diseases besides black lung. © NIOSH

The changes also call for CWP surveillance to be conducted not only by X ray, but also by lung function testing using spirometry. Robert Cohen, a professor of pulmonary medicine at Northwestern University, says this can catch damage to the airways as well as scars on the lung caused by coal mine dust exposure.

Popovich says the mining industry believes more can and must be done to protect miners from exposure. “The industry offered concrete suggestions to the Mine Safety and Health Administration during the agency’s rule-making proceeding on a new dust standard,” he says, specifically calling for mandatory participation of all coal miners in the NIOSH surveillance program and adoption of a hierarchy of progressively more protective controls to reduce miners’ exposure to respirable dust.^41^ Popovich says these suggestions were not adopted by the agency.

It’s too soon to say whether the measures that were adopted will actually cause CWP numbers to drop, but researchers hope they will. “A disease that disables around ten percent of workers would be unacceptable in any other environment,” Blackley says. “They shouldn’t have to be exposed to this risk.”
